# Multimodal analgesia versus traditional opiate based analgesia after cardiac surgery, a randomized controlled trial

**DOI:** 10.1186/1749-8090-9-52

**Published:** 2014-03-20

**Authors:** Sulman Rafiq, Daniel Andreas Steinbrüchel, Michael Jaeger Wanscher, Lars Willy Andersen, Albert Navne, Nikolaj Bang Lilleoer, Peter Skov Olsen

**Affiliations:** 1 Department of Cardiothoracic Surgery, The Heart Centre, Rigshospitalet, Blegdamsvej 9, Copenhagen O 2100, Denmark; 2 Department of Cardiothoracic Anesthesia, The Heart Centre, Rigshospitalet, Copenhagen, Denmark

**Keywords:** NSAID, Gabapentin, Multimodal, Morphine, Dexamethasone, Cardiac surgery, Postoperative pain, Analgesia

## Abstract

**Background:**

To evaluate if an opiate sparing multimodal regimen of dexamethasone, gabapentin, ibuprofen and paracetamol had better analgesic effect, less side effects and was safe compared to a traditional morphine and paracetamol regimen after cardiac surgery.

**Methods:**

Open-label, prospective randomized controlled trial. 180 patients undergoing cardiac procedures through median sternotomy, were included in the period march 2007- August 2009. 151 patients were available for analysis. Pain was assessed with the 11-numeric rating scale (11-NRS).

**Results:**

Patients in the multimodal group demonstrated significantly lower average pain scores from the day of surgery throughout the third postoperative day. Extensive nausea and vomiting, was found in no patient in the multimodal group but in 13 patients in the morphine group, p < 0.001. Postoperative rise in individual creatinine levels demonstrated a non-significant rise in the multimodal group, 33.0±53.4 vs. 19.9±48.5, p = 0.133. Patients in the multimodal group suffered less major in-hospital events in crude numbers: myocardial infarction (MI) (1 vs. 2, p = 0.54), stroke (0 vs. 3, p = 0.075), dialysis (1 vs. 2, p = 0.54), and gastrointestinal (GI) bleeding (0 vs. 1, p = 0.31). 30-day mortality was 1 vs. 2, p = 0.54.

**Conclusions:**

In patients undergoing cardiac surgery, a multimodal regimen offered significantly better analgesia than a traditional opiate regimen. Nausea and vomiting complaints were significantly reduced. No safety issues were observed with the multimodal regimen.

**Trial registration:**

Clinicaltrials.gov identifier: NCT01966172

## Background

Pain after cardiac surgery is caused by several factors; sternotomy, sternal/rib retraction, pericardiotomy, internal mammarian artery harvesting, saphenous vein harvesting, surgical manipulation of the parietal pleura, chest tube insertion and other musculoskeletal trauma during surgery.

Traditionally postoperative pain management after cardiac surgery has been based on opiate analgesics. However, opiates have some undesirable dose-related side-effects such as nausea, constipation, vomiting, dizziness, mental confusion and respiratory depression, which substantially influence patient recovery and may delay discharge after surgery [1,2]. Furthermore current evidence suggests, that poorly treated acute postoperative pain not only influences patient well-being, but also increases the risk for development of chronic pain [1,3,4].

In the last two decades, evidence-based multimodal opiate-sparing analgesia has become increasingly widespread after non-cardiac surgery [1,5]. The rationale for administering different analgesics is not only the opiate-sparing effect, but also the accomplishment of a more effective pain management, through both central and peripheral anti-nociceptive mechanisms [1,4,6].

Non-steroid anti-inflammatory drugs (NSAIDs) exert their effects by peripheral blockage of prostaglandin synthesis [1]. NSAIDs have demonstrated opiate sparing effects in randomized trials after cardiac surgery [2,7,8]. But cardiac surgeons and anaesthesiologists have had safety concerns with NSAIDs regarding renal impairment, bleeding risk and increased risk for cardiovascular death [2,9].

Gabapentin is a gamma-aminobutyric acid (GABA) analogue, and is mainly utilized for the treatment of epilepsy and neuropathic pain, but it has in recent years been used as part of multimodal regimens after non-cardiac surgery [1,10]. Gabapentin exerts its effects mainly by inhibiting central neuronal sensation [1,10]. Recent trials have examined its efficacy after cardiac surgery, but the results have been divergent [11-13]. Theoretically the addition of gabapentin to a NSAID should provide synergistic analgesic and opiate sparing effects, and indeed this combination has demonstrated some benefits in other surgical populations [1,10,14].

Dexamethasone is a corticosteroid, and therefore has anti-inflammatory properties [15,16]. Small doses of dexamethasone have in clinical trials demonstrated improved postoperative patient recovery by especially reducing fatigue, nausea and vomiting complaints [15,16]

The aim of this study was to evaluate a multimodal analgesic regimen consisting of dexamethasone, gabapentin, ibuprofen and paracetamol against an opiate based regimen of morphine and paracetamol. We hypothesized that the multimodal regimen had a better analgesic effect, reflected by an improved pain scoring, and a reduction of side-effects compared to the morphine group. Furthermore we wanted to evaluate the safety aspects of the multimodal regimen.

## Methods

### Study population

One hundred and eighty patients undergoing a cardiac procedure at our institution were randomized on the day before surgery. Study period was from March 2007 to August 2009. Inclusion criteria were: age > 18, any cardiac procedure with sternotomy, and able to give informed consent. Exclusion criteria were; cardiac surgery without sternotomy, peripheral neuropathy, neurological disease, psychiatric illness, history of GI bleeding, chronic pain (i.e. back pain, cancer, arthritis), serum creatinine >150 μmol/l, hepatic disease with elevated liver enzymes (SGPT and SGOT elevated to 1.5 times maximum normal value), allergic to study medication, alcohol abuse, abuse of narcotics or medication, pregnancy, participation in other clinical trials, insufficient language skills. In addition intensive care unit (ICU) stay for more than 24 hours was used as a pre-defined post randomization exclusion criteria, because prolonged ICU stay and ventilator treatment would interfere with study analgesic protocol.

### Ethics

The local ethics committee (Danish Research Ethics Committee) approved the study. All participants gave written informed consent before inclusion. The study was conducted in accordance with the Helsinki 2 declaration.

### Study design and randomization

Single-center, prospective randomized controlled trial, with an open-label design. Patients were approached on the day before surgery. After obtaining informed consent, the patients were randomized utilizing sequentially numbered opaque sealed envelopes to one of two groups (ratio 1:1) by the study co-ordinator.

Clinicaltrials.gov identifier: NCT01966172.

### Anaesthetic management

Identical anaesthetic protocol was used in both groups. Peripheral venous, central venous and urinary catheters were inserted. Radial artery pressure was monitored. Anaesthesia was induced with 2-5 mg of midazolam, 10 μg/kg of fentanyl and 8 mg pancuronium. After intubation anaesthesia was supplemented with isoflurane 0.4 to 1 %. Fentanyl was during surgery supplemented up to a total dose of 25 μg/kg. After finalization of extracorporal circulation (ECC) propofol infusion was started at an infusion rate of 100-200 mg/hour. The patients were subsequently transferred anaesthetized to the intensive care unit (ICU) for further stabilization and recovery. When pressure support on the ventilator was reduced to 10 cm H_2_O and Positive End Expiratory pressure(PEEP) was 5-7 cm H_2_O, 4 mg of ondasteron iv were administered, the propofol infusion was stopped and the patients were extubated.

All patients were given a single 240 mg dose of gentamicin and a 1500 mg dose of cefuroxim intravenously at initation of surgery for antibiotic prophylaxis. Cefuroxim 1500 mg three times daily was continued untill the morning of the third postoperative day.

### Surgical procedures

There was no selection for a specific type of cardiac surgery. All surgical procedures were performed through median sternotomy. Patients undergoing CABG surgery had left internal mammarian artery (LIMA) pedicled conduits harvested using a LIMA retractor. Saphenous vein grafts were harvested using a open technique in 90% of cases, and 10% were harvested with endoscope. Proximal anastomoses were performed using side clamp. Patients on ECC were heparinised to maintain an acquired coagulation time (ACT) above 480 s, and this was reversed with protaminesulphate (1:1) after finalization of ECC. Blood cardioplegia was used to induce cardiac arrest.

Two chest tubes (28 F) were placed- one mediastinally and the other in the left hemithorax- both were passed subxiphoidally. The sternotomy was closed with 8 steel wires, and the skin incision was closed with intracutaneous suturing.

### Analgesic protocol (intervention)

Pre-operatively both groups received 0.125 mg triazolam orally 1 hour before surgery.

Multimodal group: Before extubation, at the time of ondasteron administration, 30 mg of ketorolac (a NSAID) and 8 mg of dexamethason were administered intravenously together with 1 g of paracetamol rectally. In the evening of the operative day, at 10 pm 300 mg of gabapentin, 1 g of paracetamol and 1 g of the laxative magnesiumoxide were administered. From the first postoperative day through the fourth postoperative day, patients received following oral preparations; 300 mg of gabapentin twice daily, 400 mg of ibuprofen four times daily, 1 g of paracetamol four times daily, 40 mg of the proton pump inhibitor pantoprazol once daily and 1 g of magnesiumoxide once daily.

Morphine group: On arrival at ICU 5 mg of morphine iv were administered, further administrations were allowed up to a total of 25 mg. At time of ondasteron administration before extubation, 1 g of paracetamol was administered rectally. At 10 pm 1 g of paracetamol and 1 g og magnesiumoxide were administered. From the first postoperative day through the third postoperative day, patients received following oral preparations; 10 mg of morphine four times daily, 1 g of paracetamol four times daily, 40 mg pantoprazol once daily and 1 g of magnesiumoxide once daily. On the fourth day oral morphine was reduced to 5 mg four times daily.

An overview of administered analgesics is presented in Table 1.

**Table 1:** Overview of administered analgesics

	**Multimodal**	**Morphin**
Extubation	•Inj.ketorolac 30 mg iv	•Inj morphine 5-25 mg iv
	•Inj dexamethasone 8 mg iv	•Inj ondasterone 4 mg iv
		•Rectal suppositorium paracetamol 1000 mg
	•Inj ondasterone 4 mg iv	
	•Rectal suppositorium paracetamol 1000 mg	
Day 1	•Tbl Ibuprofen 400 mg x 4	•Tbl Morphine 10 mg x 4
		•Tbl Paracetamol 1000 mg x 4
	•Tbl Gabapentin 300 mg x 2	
	•Tbl Paracetamol 1000 mg x 4	
Day 2	•Tbl Ibuprofen 400 mg x 4	•Tbl Morphine 10 mg x 4
		•Tbl Paracetamol 1000 mg x 4
	•Tbl Gabapentin 300 mg x 2	
	•TblParacetamol 1000 mg x 4	
Day 3	•Tbl Ibuprofen 400 mg x 4	•Tbl Morphine 10 mg x 4
		•Tbl Paracetamol 1000 mg x 4
	•Tbl Gabapentin 300 mg x 2	
	•TblParacetamol 1000 mg x 4	
Day 4	•Tbl Ibuprofen 400 mg x 4	•Tbl Morphine 10 mg x 4
		•Tbl Paracetamol 1000 mg x 4
	•Tbl Gabapentin 300 mg x 2	
	•TblParacetamol 1000 mg x 4	

Administrations are noted by times daily with (x),, *x*2 equals twice daily and x4, four times daily. Inj = injection, iv = intravenous, tbl = tablet.

In both groups, if pain was still unacceptable for the patient, the attending nurse was allowed to administer morphine either as an injection of 5-10 mg iv or an oral dose of 10 mg as needed until pain relief. All administered doses were recorded. Also administration of extra NSAID (Ibuprofen and ketorolac) was registered.

In both groups supplementary medication for relief of nausea was 4 mg ondasteron iv up to three times daily, and/or 10 mg of metoclopramid iv up to three times daily.

### Data collection

From the evening of surgery, after extubation, and then every evening until the 4^th^ postoperative day, pain intensity was reported by the patient, utilizing the 11-point numeric rating scale (11-NRS). For clarity this is the numeric version of the more widespread visual analogue scale (VAS). The 11-NRS has been validated as a sensitive tool for assessing and scoring pain in the postoperative setting [17]. Patients reported, ‘worst pain’ experienced during the day, ‘least pain’ , ‘average pain’ and ‘current pain’ at 8 pm, all on 11-NRS. Pain status was also reported in the morning before analgesia administration; patients were asked for current pain in the sternum, and overall physical pain both on 11-NRS. Furthermore, patients in the evening reported on a questionnaire whether they had experienced any of following symptoms during the last 24 hours: nausea, constipation, vomiting, difficulty with urination, dizziness, mental confusion, difficulty concentrating and fatigue. The answers were recorded as (‘not at all’, ‘rarely’, ‘sometimes’, ‘often’ and ‘all the time’).

With respect to the secondary outcomes, all major in-hospital events (cardiac,renal,cerebral,thromboembolic,GI,sternal) were collected. MI was defined as CKMB levels over 80 Units/l in the immediate postoperative period, ECG changes and/or breast pain, coronary angiography was performed to confirm diagnosis. Re-admission to ICU and the need for inotropic support was recorded. Creatinine levels during hospitalization were recorded. The need for dialysis was determined by the attending cardiac surgeon and anaesthesiologist. Cerebral complications were recorded as transitory cerebral ischemia (TCI), if symptoms lasted less than 24 hours. Symptoms lasting more than 24 hours were classified as strokes. Incidence of GI bleeding or any other bleeding were recorded, and confirmed by gastroscopy.

### Primary outcome

1. Evaluation of analgesic effect by 11-NRS scale.

### Secondary outcomes

2. Additional analgesic consumption.

3. Hospital stay in days.

4. Evaluation of side-effects by daily questionnaire.

5. Renal complications: increase in creatinine, dialysis.

6. Cardiac complications; Myocardial infarction(MI), postoperative pericardial effusion, heart failure requiring inotropic support, atrial fibrillation.

7. Other complications: cerebral (stroke, bleeding), GI(bleeding), blood component requirements and sternal complications.

8. Death from all causes.

Outcomes 1-4 were recorded and evaluated during index hospitalization. Outcomes 5-8 were recorded for 30 days after surgery.

### Sample size

Sample size was calculated, assuming patients on a morphine regimen would have a mean NRS-11 score of 4, and patients in the multimodal regimen would have a mean of 3, with a power of 80% and significance level of 5%, each group would have to have 63 patients. Therefor, initially 75 patients were planned in each group, to account for dropouts. As the degree of dropouts due to ICU stay over 24 hours exceeded the expected, it was decided to include 90 patients in each group.

The study was primarily powered to test the primary hypothesis.

### Statistical analysis

For categorical variables chi-square tests or the Fischer’s exact test were used as appropriate, and 2-sided significance values were evaluated. For continuous variables analysis was performed with the two sample *t*-test, after ensuring normal distribution of data. Data with no normal distribution were analyzed by Wilcoxon rank sum test. Continuous variables are presented as mean±standard deviation (SD) unless otherwise noted. P < 0.05 was considered statistically significant. All P-values above 0.10 are denoted NS (non-significant) in tables. Data entry and analysis was performed blinded to group allocation. All statistical analyses were performed using SPSS statistical software v.19.0 (SPSS inc, Chicago IL).

## Results

**Figure 1:**
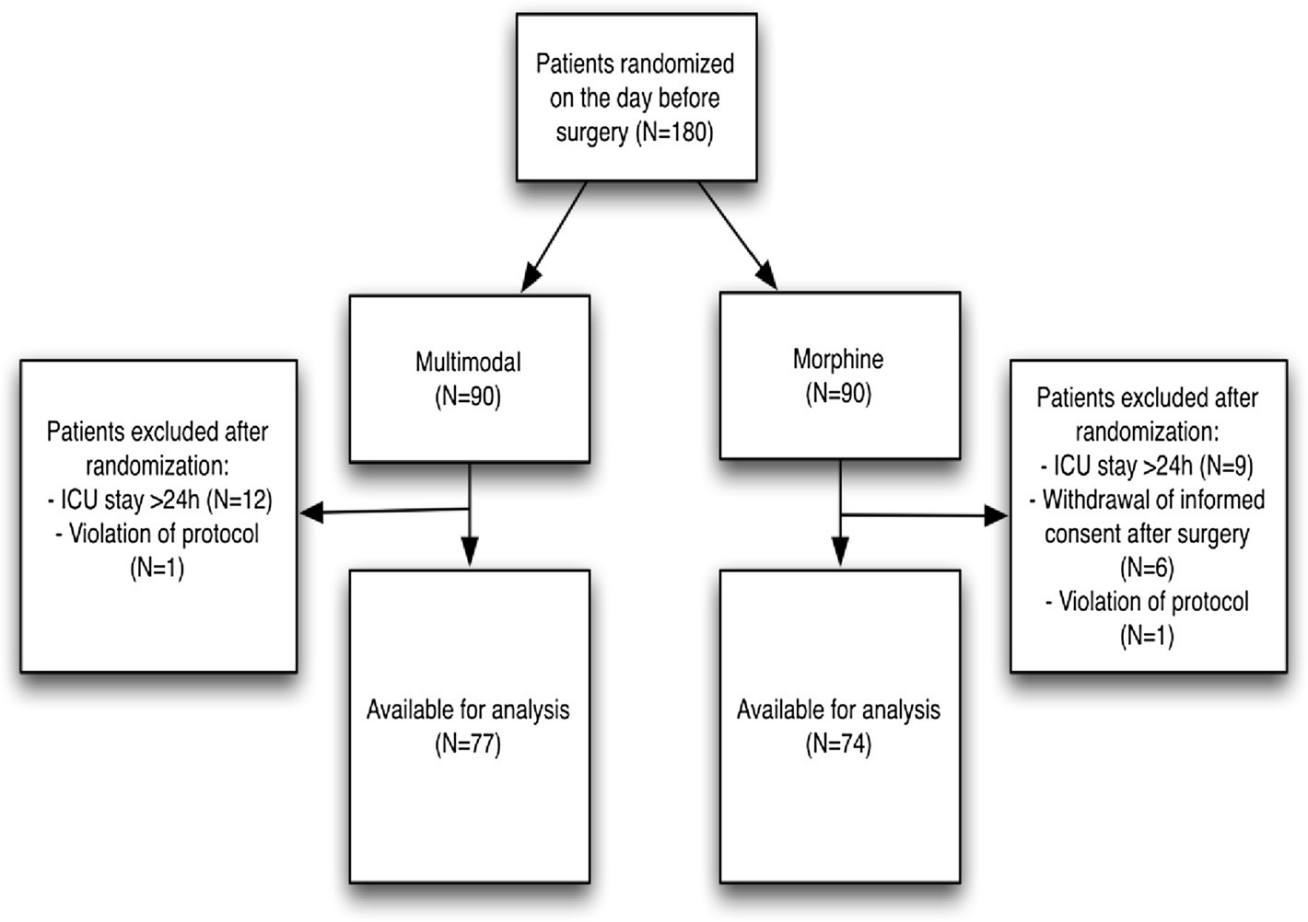
Study flow-chart.

A total of 180 patients were randomized. Twenty one patients were excluded after surgery due to prolonged ICU stay over 24 hours for different reasons i.e. re-do surgery, heart failure etc. (pre-defined exclusion criteria). Furthermore 6 patients in the morphine group withdrew informed consent after surgery, because of nausea/vomiting and reluctance to pain scoring. Two patients were excluded from further analysis because of inclusion criteria violation- both had pre-operative creatinine above 150 μmol/l - see flow chart (Figure 1). Thus a total of 29 patients were excluded from the study. Hence data from 151 patients was available for analysis.

Demographic data of these patients are presented in Table 2.

**Table 2:** Demographic data

	**Multimodal (n = 77)**	**Morphine (n = 74)**	**P-value**
Age (Mean ± SD)	62 ± 12	64 ± 13	NS
Female (n)	16	15	NS
BMI (Mean ± SD)	27.4 ± 5.1	28.1 ± 4.3	NS
Risk factors/co-morbidity (n(%))			
Hypertension	48	47	NS
Hypercholest.	55	58	NS
Diabetes	8	16	0.06(NS)
Peripheral arterial disease	8	6	NS
Previous TCI/stroke	7	6	NS
Severe COPD	5	4	NS
Alcohol (Median (min-max))	7[0-42]	6[0-42]	NS
Cardiac history (n)			
MI past (>months)	14	14	NS
MI present (<3 months)	5	4	NS
CCS	2 ± 1	2 ± 1	NS
NYHA class	2 ± 1	2 ± 1	NS
Atrial fibrillation	5	9	NS
LVEF > 50%	33	35	NS
LVEF 30%-50%	36	26	NS
LVEF <30%	8	13	NS
Euroscore (Mean ± SD	5.0 ± 2.9	5.2 ± 3.4	NS
Creatinine (μmol/J)	82.6 ± 17.0	82.7 ± 15.1	NS

BMI = body mass index, TCI = transitory cerebral ischemia, COPD = chronic obstructive pulmonary disease, MI = myocardial infarction, CCS = canadian cardiovascular society, NYHA = New york heart association, LVEF = left ventricular ejection fraction, NS = non-significant.

Operative data are presented in Table 3.

**Table 3:** Operative data

	**Multimodal (n = 77)**	**Morphine (n = 74)**	**P-value**
Types of surgery (n)			
CABG	44	42	NS
OPCAB	1	3	NS
CABG + valve	8	8	NS
Valve	18	15	NS
Other	6	6	NS
Perfusion data			
Perfusion time (min)	83.4±32.5	91.4±37.6	NS
Clamp time (min)	56.6±26.6	62.4±30.8	NS

CABG = coronary artery bypass graft, OPCAB = off pump coronary artery bypass.

NRS-11 pain scoring data are presented in Table 4. It is noteworthy that patients in the multimodal group, in all categories, except “worst pain” on day 4, had lower mean pain scores. Although not all of these differences reached statistically significance, patients scored significantly lower on average pain sensation from day 0 (day of surgery) throughout to day 3. The least pain experienced during the day was also lower in the multimodal group from day 1 to day 3.

**Table 4:** Pain scoring by 11-NRS

	**Day 0**		**Day 1**		**Day 2**		**Day 3**		**Day 4**	
	**Painscore, mean±SD**	**P-value**	**Painscore, mean±SD**	**P-value**	**Painscore, mean±SD**	**P-value**	**Painscore, mean±SD**	**P-value**	**Painscore, mean±SD**	**P-value**
**Evening scores**										
**Worst pain**										
Multimodal	4.5±2.4	NS	4.6±2.4	0.057(NS)	4.4±2.7	0.093(NS)	3.6±2.4	NS	3.9±2.5	NS
Morphine	5.0±2.4		5.5±2.4		5.3±2.1		3.9±2.3		3.5±2.2	
**Least pain**										
Multimodal	1.5±0.7	NS	1.6±1.0	0.042	1.7±2.4	0.021	1.3±0.8	0.021	1.4±1.0	NS
Morphine	1.7±0.9		2.1±1.2		2.5±1.7		1.8±0.8		1.6±0.8	
**Average pain**										
Multimodal	2.6±1.2	0.022	2.7±1.4	0.005	2.5±1.5	0.001	1.9±1.2	0.032	2.1±1.6	NS
Morphine	3.5±2.0		3.6±1.6		3.9±1.7		2.7±1.5		2.7±1.7	
**Current pain**										
Multimodal	2.5±1.8	NS	2.5±2.0	NS	2.1±1.9	NS	1.4±0.9	0.038	1.7±1.5	NS
Morphine	2.3±1.6		3.1±2.2		2.6±1.9		2.2±1.7		1.7±1.0	
**Morning scores**										
**Sternal pain**										
Multimodal	NA		NA		2.3±2.4	0.042	1.7±2.0	NS	1.6±1.7	NS
Morphine					3.1±2.5		2.0±1.8		1.6±1.9	
**Physical pain**										
Multimodal	NA		NA		2.0±2.2	NS	1.4±0.9	NS	1.3±1.7	NS
Morphine					2.3±2.4		2.2±1.7		1.6±1.8	

For each day pain scores (mean ± SD) between groups are listed. NA = Not Applicable NS = Non Significant.

Supplemental Analgesics use is presented in Table 5. For each group it is was recorded how many patients used supplementary analgesics, and what the mean consumption was in these patients for the whole study period.

**Table 5:** Supplemental analgesics

	**Multimodal**	**Morphine**	**P-value**
**Supplementary morphine**			
Number of patients	36	42	NS
Average consumption in mg (mean ± SD)	16.0 ± 17.2	21.6 ± 20.7	NS
**Supplementary ibuprofen**			
Number of patients	12	7	NS
Average consumption in mg (mean ± SD)	667 ± 394	2171 ± 2929	0.091(NS)
**Supplementary ketorolac**			
Number of patients	3	2	NS
Average consumption in mg (mean)	30	30	NS

Number of patients in each group that received additional analgesics, the consumption is for the whole study period, and only for those patients recieving additional analgesics".

Duration of hospital stay was insignificantly lower in multimodal vs. morphine patients, 7.4 days±3.3 vs. 8.2 days ±4.6, p = 0.257.

The comprehensive information collected from side-effect questionnaires demonstrated only minor non significant differences between groups, except for nausea and vomiting. Thirteen patients in the morphine group had to stop study medication due to nausea and vomiting in comparison to none in the multimodal group; 13 vs. 0, p < 0.001. Of these 13 patients 8 stopped day 1, 4 stopped day 2 and 1 patient stopped at day 3. The six patients who withdrew informed consent are not included in these numbers.

One case of involuntary muscular contractions of the upper extremities was observed in a patient in the multimodal group, and gabapentin was stopped, which lead to normalization in one day.

In-hospital events, transfusion requirements and 30-day mortality data are presented in Table 6. The patients in the multimodal group on average had a greater increase in creatinine postoperatively although this was not significant. Maximum postoperative creatinine levels data presented as median[min-max] were 92 μmol/l [65-314] in the multimodal group and 90 μmol/1 [33-411] in the morphine group. Two patients in the morphine group and 1 patient in multimodal group had to undergo hemodialysis. It is also to be noted here, that 4 patients in the multimodal group had their pain regimen cancelled by their attending surgeon because of concerns of elevated creatinine levels. Maximum postoperative creatinine in μmol/l and the day of stopping for these 5 patients were respectively: 155 on day 2, 257 on day 2, 260 on day 1 and 314 on day 3. Elevated creatinine levels did not lead to cancellation of pain regimen in the morphine group. Further analysis of creatinine data showed, that 25 patients in the multimodal vs. 14 patients in the morphine group had a increase of more than 30 percent in postoperative creatinine level compared to preoperative creatinine level, p = 0.089.

**Table 6:** In-hospital events, transfusion requirements and 30-day mortality

	**Multimodal (N = 77)**	**Morphine (N = 74)**	**P value**
**Renal complications**			NS
Max postop creatinine (μmol/1) (mean ± SD)	117.0 ± 60.9	102.3 ± 50.4	NS
Individual maximum rise in creatinine (μmol/1)	33.0 ± 53.4	19.9 ± 48.5	NS
Dialysis (n)	1	2	NS
**Cardiac complications**			
Myocardial infarction (n)	1	2	NS
Postoperative pericardial effusion (n)	1	3	NS
Readmitted to ICU for inotropic support (n)	1	1	NS
Atrial fibrillation	31	34	NS
**Cerebral complications**			
Stroke(n)	0	3	0.075(NS)
TCI (n)	0	1	NS
Involuntary movements (n)	1	0	NS
**GI complications**			
GI bleeding	0	1	NS
**Sternal complications**			
Sternal dehiscence	1	1	NS
Sternal infection	1	2	NS
**Transfusion requirements**			
RBC (units)	1.21 ± 2.03	1.17 ± 2.10	NS
FFP (units)	0.31 ± 0.90	0.26 ± 0.84	NS
PC (units)	0.17 ± 0.48	0.10 ± 0.42	NS
**Postoperative antiplatelet/anticoagulation therapy**			NS
Aspirin	63	57	NS
Clopidogrel	2	4	NS
VKA	20	19	NS
**Mortality**			
30 day mortality	1	2	NS

ICU = intensive care unit, RBC = packed red blood cells, FFP = fresh frozen plasma, PC = platelet concentrates, VKA = vitamin K antagonist.

## Discussion

The present study is the first to compare a multimodal regimen consisting of dexamethasone, gabapentin, ibuprofen and paracetamol against a traditional opiate based regimen (morphine and paracetamol) after cardiac surgery.

Our results show that patients in the multimodal group had significantly lower “average pain” scores from the day of surgery to the third postoperative day. Furthermore, additional morphine requirements and hospitalization demonstrated a non-significant trend towards improved outcome in the multimodal group.

These findings are in concordance with findings in other surgical populations, which have shown improved pain relief with a multimodal approach [1,14,18].

Equally important as pain relief, is the extent of side-effects from the instituted analgesic regimen. In our study 13 patients in the morphine group experienced nausea and vomiting, to such a degree, that they had to discontinue their analgesic regimen. It is to be noted, that morphine does have a dose-related side-effect profile, and it can be argued that we were not able to titrate morphine levels down as we would have ie. if we had used patient administered morphine pumps, when patients experienced side-effects. But nonetheless none of the patients in the multimodal group had such extensive nausea and vomiting symptoms. This was a very convincing and important finding of this study. Single dose dexamethasone has previously been shown to significantly reduce postoperative nausea and vomiting after cardiac surgery, and improve quality of recovery [15,16]. Also the use of NSAIDs instead of opiates after cardiac surgery has been demonstrated to significantly reduce occurrence of nausea and vomiting [2]. However other studies have failed to demonstrate that NSAIDs reduce nausea and vomiting after cardiac surgery[7-9]. Gabapentin on its own has in some studies demonstrated an anti-emetic effect, but these findings have not been confirmed in patients undergoing cardiac surgery [3,10,12].

In the present study, renal complications were not significantly higher in the multimodal group. But there was a trend towards patient in the multimodal group having greater increase in creatinine levels postoperatively. Furthermore, 4 patients in the multimodal group had their analgesic regimen stopped by the attending surgeon because of concerns of rising creatinine levels. It can be argued that our study lacked power to demonstrate a significant difference in creatinine level elevations. However, a review published by Acharya and Dunning in 2010 concluded that “..NSAIDs are not associated with an increased risk of renal failure after cardiac surgery when administered at optimal ‘renal’ doses, within early postoperative settings, to patients at low-risk of renal dysfunction in whom NSAIDs are not contraindicated.”[19]. Our results support this conclusion, but encourage clinicians to evaluate postoperative creatinine levels regularly when on a NSAID/gabapentin regimen. This is in fact the current practice at our institution, where all patients postoperatively receive a multimodal analgesic regimen unless it is contraindicated. Creatinine levels are checked on day 1,2 and 4 postoperatively

Regarding cardiac and cerebral complications, there were no significant differences between groups. A previous large-scale multicenter study demonstrated a greater incidence of cardiovascular events in patients receiving COX-2 inhibitors for 10-14 days [9]. Ketorolac and ibuprofen are the NSAIDs utilized in our multimodal regimen, these are both non-selective NSAIDs, and our results do not support the occurrence of more cardiovascular events in this group. On the contrary patients in the morphine group in our study suffered more thromboembolic complications in crude numbers. A recently published retrospective database study raised concern of NSAIDs as a whole being associated with increased deaths and recurrent MIs in a population with previous MI (not a surgical population) [20]. Interestingly the authors found that ibuprofen usage below 7 days, was not associated with increased death and MI [20]. It can be speculated that the lower incidence of thromboembolic complications in the multimodal group in our study, was due to the antithrombotic effect of the NSAIDs [21]. This effect may even be more important after cardiac surgery, as cardiac sugery has been shown to induce hypercoagulability and that hypercoagulability is associated with an increased incidence of thromboembolic complications and death after cardiac surgery [22,23]. This is speculative and future studies must elucidate this point.

### Limitations

The open label design and lack of placebo usage in this study are obvious limitations, we have tried to compensate for these by doing data entry and data analysis blinded to group allocation.

It can be argued that comparison of a whole multimodal regimen against a morphine regimen, does not pinpoint the effects of the each drug in the multimodal group, but we believe this to be a power of this study. Because, as we demonstrate, the multimodal regimen, does provide better analgesia with less side-effects after cardiac surgery, and can be used in a clinical setting as described here.

## Conclusions

In patients undergoing cardiac surgery, a multimodal regimen consisting of dexamethasone, gabapentin, ibuprofen and paracetamol offered better analgesia than a regimen consisting of morphine and paracetamol. Furthermore, nausea and vomiting complaints were reduced significantly in the multimodal group.

Although there was a non-significant increase in individual creatinine levels in the multimodal group, no safety issues regarding dialysis, MI, heart failure, stroke, GI bleeding and sternal complications and 30-day mortality were observed.

## Abbreviations

CABG: Coronary artery bypass grafting; CKMB: Creatine kinase MB; ECC: Extra corporal circulation; ECG: Electrocardiogram; GI: Gastro-intestinal; ICU: Intensive care unit; Iv: Intravenous; LIMA: Left internal mammary artery; MI: Myocardial infarction; NSAID: Non steroid anti inflammatory drug; NRS: Numeric rating scale; Sc: Subcutaneous; VAS: Visual analogue scale.

## Competing interests

The authors declare no conflict of interests.

## Authors’ contributions

SR: collected data, performed data analysis, drafted the manuscript. DAS: participated in designing the study, aided in data analysis and drafting of manuscript. MW: participated in designing the study, aided in data collection, and drafting the manuscript LWA: participated in designing the study and drafting the manuscript. AN: participated in study design, data collection, and aided in data analysis. TNL: participated in study design, data collection and aided in data analysis. PSO: Participated in study design, aided in data analysis and drafting of the manuscript. All authors read and approved the final manuscript.

## Study site

Department of Cardiothoracic Surgery, The Heart Centre, Rigshospitalet, Copenhagen University Hospital, Blegdamsvej 9, 2100 Copenhagen O, Denmark

Meeting presentation: The results have been presented at the annul meeting of the Danish Thoracic Surgery Society 2012 (first price winner), and as oral presentation at the annual meeting of the Scandinavian Association for Thoracic Surgery on the 17^th^ of August 2012 in Vilnius, Lithuania (under the award winning symposium).
